# Effects on Biochemical Parameters and Animal Welfare of Dusky Grouper (*Epinephelus marginatus*, Lowe 1834) by Feeding CTX Toxic Flesh

**DOI:** 10.3390/ani14121757

**Published:** 2024-06-11

**Authors:** Yefermin Darias-Dágfeel, Andres Sanchez-Henao, Daniel Padilla, María Virginia Martín, María José Ramos-Sosa, Paula Poquet, Michelle Barreto, Freddy Silva Sergent, Salvador Jerez, Fernando Real

**Affiliations:** 1 Division of Fish Health and Pathology, University Institute of Animal Health and Food Safety (IUSA), University of Las Palmas de Gran Canaria, 35416 Arucas, Spain; yeferminjesus.darias@ulpgc.es (Y.D.-D.); daniel.padilla@ulpgc.es (D.P.); maria.ramossosa@ulpgc.es (M.J.R.-S.); paula.poquet@ulpgc.es (P.P.); freddy.silva@ulpgc.es (F.S.S.); fernando.real@ulpgc.es (F.R.); 2 Oceanographic Centre of Canary Islands, Spanish Institute of Oceanography, Spanish National Research Council (IEO-CSIC), C. Farola del Mar 22, San Andrés, 38180 Santa Cruz de Tenerife, Spain; virginia.martin@ieo.csic.es (M.V.M.); michelle.barreto@ieo.csic.es (M.B.); salvador.jerez@ieo.csic.es (S.J.)

**Keywords:** *Epinephelus marginatus*, ciguatera poisoning (CP), ciguatoxins (CTXs), animal welfare, biochemical parameters, feeding exposure

## Abstract

**Simple Summary:**

Ciguatera poisoning is a foodborne disease caused by the consumption of fish. Ciguatoxins are toxins produced by dinoflagellates. These microalgae attached to macroalgae are consumed by herbivorous fish, which are the prey of carnivorous fish that cause food outbreaks. The dusky grouper is a species capable of bioaccumulating ciguatoxins in its tissue and causing ciguatera in humans. This experiment consisted of studying the exposure of ciguatoxins in grouper through their diet, as occurs in the wild, through flesh naturally contaminated with ciguatoxins. This study of dietary exposure of ciguatoxins in grouper fish has allowed us to know the cytotoxic effects of the diet in this species and the fish’s behavior against this toxic diet throughout the study time.

**Abstract:**

Ciguatera is a foodborne disease caused by ciguatoxins (CTXs), produced by dinoflagellates (genera *Gambierdiscus* and *Fukuyoa*), which bioaccumulate in fish through the food web, causing poisoning in humans. Currently, the physiological mechanisms of the species with the highest amount of toxins in their adult stage of life that are capable of causing these poisonings are poorly understood. Dusky grouper (*Epinephelus marginatus*) is a relevant fishing species and is part of the CTX food chain in the Canary Islands. This study developed an experimental model of dietary exposure featuring adult dusky groupers with two diets of tissue naturally contaminated with CTXs (amberjack and moray eel flesh) with two different potential toxicities; both groups were studied at different stages of exposure (4, 6, 10, 12, and 18 weeks). The results showed that this species did not show changes in its behavior due to the provided feeding, but the changes were recorded in biochemical parameters (mainly lipid and hepatic metabolism) that may respond to liver damage and alterations in the homeostasis of the fish; more research is needed to understand histopathological and cytotoxic changes.

## 1. Introduction

Ciguatera poisoning (CP) is caused by the consumption of fish that are naturally contaminated by ciguatoxins (CTXs), which are produced by dinoflagellates of the genus *Gambierdiscus* and *Fukuyoa.* In the Canary Islands, cases of ciguatera poisoning have been reported since 2004 [1]. These dinoflagellates are benthic microalgae epiphytes of other macroalgae, which produce ciguatoxins (CTXs), maitotoxins (MTXs), and other secondary toxic metabolites [2], which enter the food web through filter-feeding organisms and herbivores. Currently, there are 16 *Gambierdiscus* species described [3,4,5]. The Canary Islands have been characterized as a hotspot region for the diversity of *Gambierdiscus* species as at least six different species have been reported so far [6,7]. Typically, these species were considered endemic to tropical regions, but the warming of waters in more temperate regions has caused subtropical regions such as the Canary Islands to be considered endemic for these microalgae [7,8]. At the molecular level, CTXs act by activating voltage-gated sodium channels (VGSCs) at the intracellular level, which causes sodium entry into the cell, increasing the repetitive actuation of action potentials and causing cell death failure [9,10]. Ciguatera poisoning is characterized by the development of digestive and cardiovascular signs, as well as neurological symptoms that can last weeks, months, and even years. There is no specific treatment for this food poisoning [3,11,12].

Before the cases of ciguatera in regions such as the Canary Islands [1], the areas assumed to be endemic for ciguatera were the tropical and subtropical regions of the planet. Ciguatera affects between 25,000 and 50,000 people per year worldwide, although the incidence could be higher due to misreporting of cases. In fact, it is estimated that only 10% to 20% of ciguatera cases are reported to public health and other authorities. Carnivorous fish are typically involved in ciguatera cases, being responsible for 68% of cases in French Polynesia and 85% in New Caledonia [13]. The most commonly implicated fish are barracuda, red snapper, grouper, amberjack, sea bass, surgeonfish, and moray eel. Additionally, there is no storage, preparation, or cooking procedure that can destroy the toxin [3,9,10].

Tissue bioaccumulation of toxins and subsequent biomagnification is a central principle of the CTX food web transfer underlying ciguatera. But there is conflicting evidence about the level in the food web at which accumulated toxins become dangerous to both fish and humans [4,10,14]. The progression of CTXs through the food chain is a significant concern for human health. Initially, herbivorous fish ingest these toxins while feeding on contaminated macroalgae. Subsequently, carnivorous fish may consume these herbivores, leading to the bioaccumulation of CTXs in higher-order carnivores. As CTXs move up the food chain, they may undergo biotransformation and accumulate to hazardous levels for human consumption [3,4,11,15]. The transfer of CTXs from the prey to the predator has been demonstrated by feeding experiments, even when using flesh from a high-order carnivorous to feed omnivorous fish [16,17,18,19,20]. Research indicates that fish metabolism plays a role in increasing the toxicity of CTXs. This metabolism attempts to oxidize the CTXs into other molecules to reduce its toxicity. However, this process inadvertently results in the formation of more potent CTX congeners or an increase in toxicity [9,10].

Despite the complexities of bioaccumulation and biotransformation, it is recognized that both small fish and herbivorous fish can pose a significant risk to human health if they contain elevated levels of CTXs [3]. Marine invertebrates have also been reported as new vectors of ciguatera poisoning in regions of the Pacific and the Caribbean, with CTXs in sea urchin (*Diadema antillarum*), octopus (*Octopus cyanea*), giant clam (*Tridacna maxima*) and lobster (*Panulirus penicillatus*) [11,13] being detected. Therefore, monitoring and regulating the consumption of fish from potentially contaminated areas are essential measures for mitigating the risk of ciguatera poisoning in humans [8,11,12].

There are several studies of dietary exposure to ciguatoxins that try to determine the effects of these powerful toxins in fish, how they bioaccumulate in tissues, and the molecular transformation of the toxins after ingestion of fish [21,22,23,24,25,26]. However, experimental models have been developed that try to imitate exposure to CTXs in the environment with some species that are not within the ciguatoxin cycle, resulting in the development of symptoms in some cases [18,20,23,24]. Erratic swimming, lethargy, and lack of appetite have been reported in goldfish (*Carassius auratus*) exposed to CTXs [18,20], and in marine medaka (*Oryzias melastigma*), it has been observed how the toxins affect reproductive and embryonic development, negatively affecting the growth of fish in their first stage of life [23,27,28]. Other experimental models have studied the damage from the toxins over the months at a hepatotoxic level, applying histology to the possibly damaged tissues [26,29]. Hematological parameters and blood chemistry are of great interest in determining the health status and metabolic balance in wild and intensively farmed fish due to stress or disturbances in aquatic ecosystems [30]. However, there is not much information about how these toxins affect the behavior of an adult species that is exposed naturally, nor about their physiological response to toxins and the possible adaptations of the organism.

Dusky grouper (*Epinephelus marginatus*) holds significant importance in the Canary Archipelago due to its fishing interest; it is of high interest to professional and recreational fishermen throughout the northeast coast of the Atlantic Ocean, where this species from the Serranidae family can be found [31,32]. The dusky grouper is a large carnivore capable of feeding on large crustaceans, fish, and molluscs, with a sedentary behavior that can maintain a long period of time in the same area [32]. It is at the top of the food chain for the CTX cycle in the Canary Islands. As a large predatory species, it has relevance in the balance of ecosystems ranging from 10 to 250 m deep in rocky or abrupt bottoms [33]. As a result of their high fishing interest for professional and recreational fishermen, grouper populations have decreased over the years until being declared a vulnerable species by the International Union for Conservation of Nature (IUCN) [34]. This loss of biomass of the species has generated interest in the production of this species in aquaculture around the world to develop strategies that improve the health of populations in ecosystems [35]. This relevance amplifies given its position within the natural cycle of ciguatoxin (CTX) bioaccumulation and transformation in the trophic web, as evidenced by the literature and numerous ciguatera poisoning cases in the Canary Islands [36,37]. To date, the epidemiological surveillance service for ciguatera poisoning conducted by the Canary Islands Government has registered a total of 21 outbreaks from 2008 to 2022, of which 4 outbreaks were caused by *E. marginatus* consumption, occurring from 2012 to 2017. This risk of intoxication is controlled by the Ciguatera Control Program in the Canary Islands, in which all groupers weighing more than 12 kg are analyzed before being sold; between 2017 and 2021, a percentage of 22.81% of groupers positive for CTX was detected [36].

The aim of the present study is to analyze the main biochemical and morphometric changes in dusky grouper (*Epinephelus marginatus*), caused by ingestion of ciguatoxic flesh, in order to provide enough information about how CTXs affect naturally exposed carnivorous fish. Secondary to this, we aim to provide information on the physiological and biochemical processes that can occur in adult specimens of this fish species after a prolonged period of CTX ingestion.

## 2. Materials and Methods

### 2.1. Fish and Maintenance Conditions

Dusky grouper (*E. marginatus*) is a valuable fish species for fisheries in Canary Islands [33] and also has environmental concern due to its wild population decrease [33,34]. Additionally, is not a common cultivable grouper species; in fact, it is still being studied to find the most appropriate parameters and systems for cultivating it. The latter is the reason why the Spanish Oceanographic Institute (IEO-CSIC) from Tenerife (Canary Islands, Spain) had some adults of these animals born in captivity [38]. The IEO-CSIC kindly provided a total of 16 adult dusky groupers (*Epinephelus marginatus*) born in 2015 at their experimental culture facilities, with an average weight and length of 1480.3 ± 463.9 g and 41.1 ± 4.6 cm, respectively. The fish were individually placed into 16 indoor 1 m^3^ cylindrical tanks (1 fish per tank) and were maintained with constant water exchange (15 L/min) and aeration to ensure an adequate percentage of oxygen saturation (68.4 ± 3.9%) (Oximeter, Hannah Instruments, Villafranca Padovana, Italy) under natural conditions of water salinity and seawater temperature (21.8 ± 1.5 °C), pumped using an open circulation system. Fish were exposed to a natural photoperiod; to ensure greater welfare, half of the tank was covered with a mesh to reduce light intensity (100 lx) (Luxmeter HIBOK, Madrid, Spain). Groupers were fed 6 days a week at a ratio of 1.3% of their biomass during the experiment. Before conditioning, the fish for the experiment have been fed commercial food and frozen mackerel 3 times per week at 3% biomass.

### 2.2. Experimental Design

The experimental design was previously approved by the Ethics Committee for Animal Welfare of Centro Oceanográfico de Canarias-Instituto Español de Oceanografía (IEO-CSIC) (1236/2022) and, finally, authorized by the Department of Agriculture, Livestock, Fisheries and Water of the Canary Islands Government.

The design consisted of two groups of fish, experimental and control, and each group was divided into two subgroups: the first subgroup was fed ciguatoxic amberjack flesh (*n* = 6), referred to in this study as group A, and the second subgroup was fed ciguatoxic moray eel flesh (*n* = 4), referred to as group B. The control group was also divided into two subgroups, both fed amberjack and moray eel flesh that was negative to CTX-like toxicity (*n* = 2 for each group), respectively. Additionally, one fish that was fed commercial food and another one fed frozen mackerel were sampled on day 0 as the original controls of this study. Finally, experimental feeding of fish to toxic and non-toxic flesh lasted 18 weeks.

The number of fish was adjusted to the minimum necessary in accordance with experimental animal welfare standards so as to obtain useful results that give an idea of how CTXs affect adult dusky groupers exposed to the toxins and so as not to misuse the specimens; currently this is the most efficient way to observe differences.

### 2.3. Fish Conditioning

The specimens had to increase the frequency of intake food, the new diet, and adapt to their new location in individual tanks. This conditioning was a phase prior to the experiment; non-toxic food was provided, and the frequency of food intake progressively increased, starting from 3 days a week to daily feeding from Monday to Saturday. The flesh used in this phase corresponds to each experimental group: non-toxic moray eel flesh and non-toxic amberjack flesh, previously analyzed by an N2a cell-based assay (CBA). This adaptation period lasted for 30 days until the fish assimilated the total food ration throughout the week.

### 2.4. Food Preparation

The experimental feeding of the groupers consisted of different homogenate flesh portions from 18 amberjacks (for group A) as well as 8 portions of moray eel flesh (for group B). All specimens were captured in the Canary Islands waters, and the flesh was collected by the division of Fish Health and Pathology, University Institute of Animal Health and Food Safety (IUSA-ULPGC). Flesh homogenate was prepared by mixing only with raw flesh once the skin and connective tissue had been removed. They were stored frozen in plastic bags until one day before preparing the ration corresponding to each fish.

The presence of CTXs in the amberjack and moray eel specimens was previously determined by CBA individually before producing the flesh homogenate. Once each flesh homogenate was created, the toxic potential was assessed by CBA; the resulting toxicities were 0.109 ± 0.0027 ng Eq. CTX1B/g of flesh for amberjack homogenate and 0.023 ± 0.0001 ng Eq. CTX1B/g of flesh for moray eel homogenate. The cytotoxicity assay was conducted as previously described by Caillaud et al. 2012 [39]. The liquid chromatography–mass-spectrometry (LC-MS/MS) analysis, carried out at the Institute of Agrifood Research and Technology (IRTA), confirmed C-CTX1 as the major analog present in the amberjack flesh homogenate; however, the low toxicity presented in moray eel homogenate avoided the determination of CTXs by chromatographic analysis.

### 2.5. Sampling

**Figure 1:**
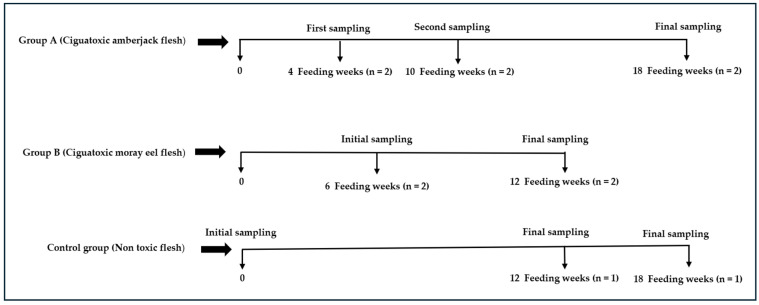
Temporal description of sampling and number of fish sampled in each experimental group.

Fish sampling was established using weeks of dietary exposure and two replicates for each sampling (*n* = 2) (Figure 1). An initial sampling was carried out in each dietary group, the commercial diet and mackerel, from the conditioning period. Experimental group A was sampled at week 4, 10, and 18, and group B was sampled at week 6 and 12. Control groups A and B were sampled on day 0 and at week 18 and 12, respectively (Table 1).

To perform the sampling, fish were euthanized with an overdose of tricaine methanesulfonate (MS-222) (30 mg L^−1^). After confirming the absence of vital signs, the corresponding sampling was carried out. The blood samples were taken from the caudal vessels using heparinized syringes for hematological and plasma biochemical analysis.

During the study, specific growth rate (SGR, % day^−1^), feed intake (FI, % body weight), condition factor (CF, g cm^−3^), hepatosomatic index (HSI%), gonadosomatic index (GSI %), and weight gain (g) were calculated as below:

$$\mathrm{SGR}=\frac{\ln\;\mathrm{final}\;\mathrm{Body}\;\mathrm{weight}\;\left(g\right)-\ln\;\mathrm{initial}\;\mathrm{Body}\;\mathrm{weight}\;(g)}{\mathrm{days}}\left(\times 100\right)$$

$$\mathrm{FI}=\frac{\mathrm{Feed}\;\mathrm{consumption}\;\left(g\right)}{\mathrm{average}\;\mathrm{biomass}\;\left(g\right)\times \mathrm{days}}\left(\times 100\right)$$

$$\mathrm{CF}=\frac{\mathrm{Body}\;\mathrm{weight}\;\left(g\right)}{\;{\mathrm{Total}\;\mathrm{lenght}\;(\mathrm{cm})\;}^{3}}\left(\times 100\right)$$

$$\mathrm{HSI}=\frac{\mathrm{Liver}\;\mathrm{weight}\;(g)}{\mathrm{Body}\;\mathrm{weight}\;(g)}\left(\times 100\right)$$

$$\mathrm{GSI}=\frac{\mathrm{Gonads}\;\mathrm{weight}\;(g)}{\mathrm{Body}\;\mathrm{weight}\;(g)}\left(\times 100\right)$$

$$\mathrm{Weight}\;\mathrm{gain}\;\left(g\right)=\mathrm{final}\;\mathrm{Body}\;\mathrm{weight}-\mathrm{initial}\;\mathrm{Body}\;\mathrm{weight}$$

### 2.6. Hematology and Blood Biochemical Analysis

The blood samples collected were analyzed to determine hematocrit (HTC) using micro-hematocrit capillaries filled with blood and centrifuged at 12,000 rpm for 5 min, and the results were expressed as the percentage (%) of total blood volume. Red blood cells (RBCs) and white blood cells (WBCs) were counted using a Neubauer hemocytometer using Natt and Herricks (1952) solution (1/100).

The remaining blood sample of each specimen was centrifuged at 15,000 rpm for 20 min, and the plasma obtained was frozen (−80 °C) for further analysis. The plasma biochemical parameters analyzed were glucose (GLU), protein (PROT) lactate (LACT), triglycerides (TRIs), cholesterol (CHOL), alkaline phosphatase (ALP), and aspartate transaminase (AST/GOT) levels, measured in duplicates by enzymatic colorimetric assays, and the laboratory tests were carried out using commercial tests (Byosistems, Barcelona, Spain). The assays were performed using a Power Wave microplate spectrophotometer (Bio-Tek Instruments, Winooski, VT, USA).

### 2.7. Visual Behavior Analysis

During the last 5 weeks of the study, fish from the control and experimental group A were recorded using a waterproof camera. The recordings were performed at different times of the day, one in the mid-morning (11:00 a.m.) after feeding and another in the afternoon (16:00 p.m.), with a duration of 5 min each. A count of the movements of the pectoral and dorsal fins was performed in addition to the analysis of respiration through opercular movement. The time of each recording started two minutes after introducing the camera in the tank.

### 2.8. Statistical Analysis

Statistical results are expressed as the means ± standard deviation. Data were transformed when needed to fulfil the assumptions of normality or equal of variance. Differences between treatments were determined by the non-parametric Kruskal–Wallis multiple comparison test. In all the statistical tests used, differences were considered significant at *p* < 0.05. The statistical analysis was performed using the SPSS statistical package (Version 21.0) for the Windows software package.

## 3. Results

### 3.1. Food Intake

**Figure 2:**
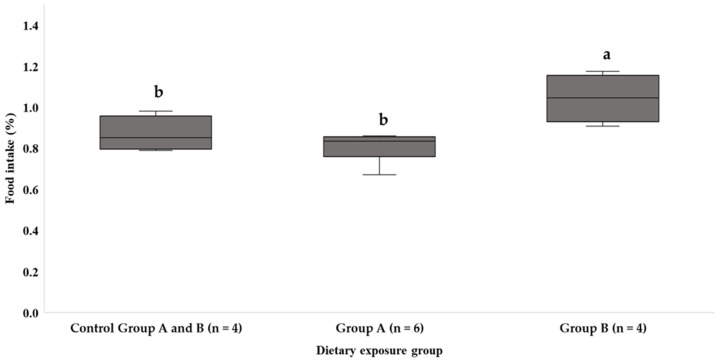
Percentage of intake consumed in each dietary exposure group throughout the study. Different letters indicate significant differences between the groups. Different letters between groups indicate significant differences (*p* < 0.05).

During the study, experimental group B showed a significantly higher intake compared with the other dietary groups (1.12 ± 0.47) in the overall period of study. Furthermore, throughout the experiment, the average intake was higher in the ciguatoxin diet groups (1.03 ± 0.13), but no significant differences were detected in this case (Figure 2). Regarding intake between CTX accumulation periods, there were no significant differences between samplings.

Experimental group A, fed a toxic amberjack diet, showed a similar intake to the control group. In these two groups, the control and experimental groups (A and B), the diet decreased throughout the trial, but the decrease in the intake of the experimental diet in group A was significant. The intake of experimental group B was also similar regardless of its toxicity (Table 2).

### 3.2. Biometric Indexes

Regardless of the feeding group, no significant differences were found in the specific growth rate and condition factor during the experiment. However, group B showed the highest average value of the specific growth rate as well as the highest condition factor and average weight gained compared with the rest of the groups during the study time; the specific growth rate of specimens fed toxic amberjack (group A) decreased by week of exposure. (Table 3). Between the toxic and non-toxic feeding groups, it was observed that the fish in the control group showed the lowest SGR (0.14 ± 0.16) in comparison with the other groups.

Regarding the hepatosomatic index, the highest mean HSI was recorded in group B compared with the other groups. Between the feeding periods, the highest average of HSI and GSI was detected in the sampling of group A at week 18 in the experimental group. However, in group B, both the toxic and non-toxic group showed the lowest mean GSI value compared with the other dietary groups. No significant differences were detected in these groups (Table 4).

### 3.3. Hematocrit and Blood Biochemistry

The hematocrit values were found to be higher in the last sampling of experimental group A than the rest of the dietary groups. However, the average values of red blood cells and white blood cells in this group were lower in the final sampling compared with the previous ones at week 4 and week 10. But no significant differences were found in these comparisons.

**Figure 3:**
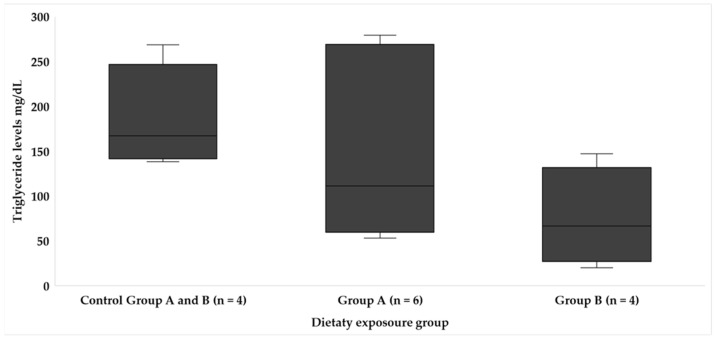
Blood triglyceride levels in each dietary exposure group throughout the study.

**Figure 4:**
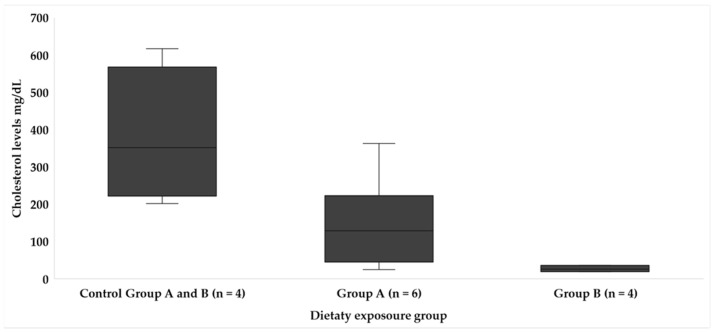
Blood cholesterol levels in each dietary exposure group throughout the study.

The levels of plasma biochemical parameters, GLU, PROT, LACT (Table 5), TRIG (Figure 3), and CHOL (Figure 4), analyzed did not show statistical differences between dietary regimes; however, some tendencies were observed in LACT levels, with the highest measurements being recorded in the fish from experimental group A at the end of the exposition period. Moreover, despite no statistical significance being found in the average level of TRIG and total CHOL between fish from the experimental groups (A and B) and control groups (*p =* 0.057), the average level tends to be lower in those fed a toxic diet. Additionally, the TRIG levels were similar between the sampling periods of both types of feeding in fish exposed to toxic feeding.

**Figure 5:**
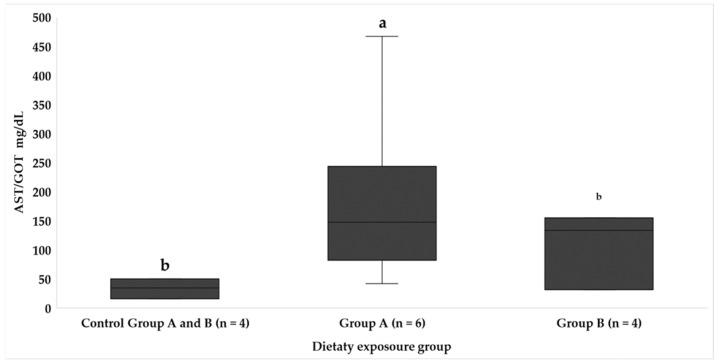
Blood AST/GOT levels in each dietary exposure group throughout the study. Different letters between groups indicate significant differences (*p* < 0.05).

**Figure 6:**
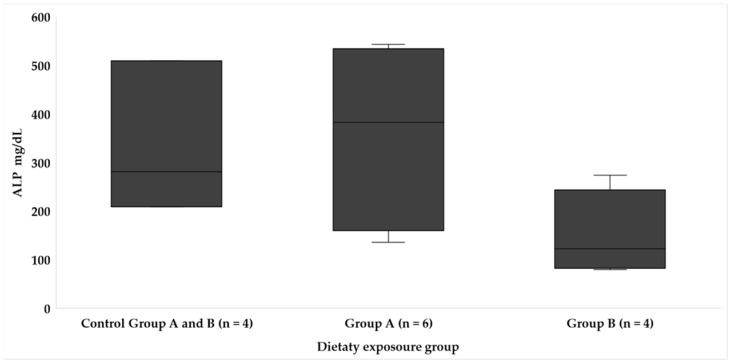
Blood ALP levels in each dietary exposure group throughout the study.

Concerning the levels of liver enzymes (ALP and AST/GOT), significant differences were found only in the enzyme AST (Figure 5) (*p* < 0.05), whose levels were significantly higher in fish from experimental group A. Additionally, although no significant differences were found in the assessment of the enzyme ALP (Figure 6), the highest average values were also determined in the fish from group A fed the most toxic diet. It is important to note that the increase in these values was progressive as the feeding time increased. In relation to group B, the levels of the liver enzymes monitored in this study did not show statistically significant differences between the experimental and control group within this category.

### 3.4. Visual Analysis

The analysis of the recordings conducted along the experiment, looking for abnormal behavioral signs such as swimming patterns, food refuse or attraction, abnormal interactions, and respiratory patterns, was carried out daily. However, no abnormal signs were detected in any of the recorded fish. Moreover, the counting of the pectoral and dorsal fin movement also did not show significant differences among the studied fish.

## 4. Discussion

The suitability of the specimens selected to perform this study attends to a sum of conditions and necessities. On the one hand, dusky grouper (*E. marginatus*) is a valuable fish species for its fishing interest in the Canary Archipelago [32], and it becomes more relevant as this species is within the natural cycle of bioaccumulation and transformation of CTXs in the trophic web, as demonstrated by the consulted bibliography and numerous ciguatera poisoning outbreaks in the Canary Islands [3,4,36,37]. On the other hand, CTXs are a group of lipophilic polyether compounds that could cause intoxications at low concentrations; the Food and Drug Administration (FDA) and European Food Safety Authority (EFSA) established a 0.01 ppb of Pacific CTX1B for safety consumption, although this level is increasingly questioned and may decrease (FAO and WHO, 2020). Moreover, these toxins have a long permanence in fish tissues for months or even years in fish that are naturally exposed [10,40], meaning that in experimental exposure studies, it becomes necessary to ensure that the fish selected have never been exposed to ciguatoxins; usually, this leads to choosing freshwater fish species or, in the case of seawater fish species, the use of cultured post-larvae or juvenile fish [17,18,21,22], with the given susceptibility associated with age that this may entail. We have obtained these fish specifically for this experiment, with a limited number of samples at a statistical level; however, it is sufficient for establishing trends that allow us to understand the pathophysiological effect of CTXs in naturally exposed fish.

### 4.1. Food Intake

This study delves into the dietary patterns of different experimental groups fed amberjack and moray eel flesh, referred to as group A and B, respectively. It is well known that these two kinds of fish are also part of the natural cycle of ciguatoxin bioaccumulation and transformation in the trophic web, and they have been reported as species causing ciguatera outbreaks [41,42,43]. Group B exhibited a notably higher intake compared with other dietary groups (group A and control group) throughout the entire study period. Also, elevated average intake levels in the ciguatoxin (CTX) diet groups throughout the experiment were observed, although no statistically significant differences were detected in this regard, as illustrated in the Figure 2.

The food preference of group B over group A can be explained by the feeding patterns of groupers, and this result could be related to the composition and palatability of the food provided. Moray eel is a common prey of dusky groupers in their natural environment, while amberjack meat is not. It is known that fishermen use moray flesh as bait to catch groupers [44], Also, groupers inhabit dark environments and rocky caves that are also frequented by moray eels [32,33]. Additionally, in the Canary Islands, the presence of a CTX-positive black moray (*Muraena augusti*) found in the stomach content of a ciguateric dusky grouper corroborates the interaction between both species and their relevance in the maintenance of ciguatoxins in the marine environment [44]. One intriguing aspect of the findings is the absence of statistical differences in intake throughout the study of CTX accumulation. This implies that, despite potential variations in the levels of ciguatoxin accumulation, the dietary habits and intake remained relatively stable. This observation raises questions about the adaptability or tolerance of the subjects to varying CTX levels in their diet, especially in piscivorous fish, located at the top of the food web, which are capable of feeding on a greater amount of zooplanktivorous fish [34,35]. This fact, in which food intake remained constant in the group B toxic diet, does not seem to occur in group A, which was also fed toxic amberjack flesh and whose intake decreased progressively. These results of our study coincide with other experimental models in which intake progressively decreases with the feeding of toxic flesh [18,19]. However, there are few studies of dietary exposure to ciguatoxin using an experimental model of a carnivorous fish fed toxic tissues [17,21,22]. In the experimental model carried out by Li et al. [22], the toxins CTX1B, CTX2, and CTX3 were isolated and purified from the viscera of moray eels and added to the food of orange-spotted groupers (*Epinephelus coioides*); in this study, the intake was maintained at a constant during the feeding period. The authors did not detect mortality, and their results focused on the assimilation and purification of the toxin in the tissues and did not comment on the appearance of symptoms in the fish due to the accumulation. In some experimental models, some researchers have used cultures of maitotoxin-producing microalgae such as *Gambierdiscus australes* injected into tissue for use as food; in that study, snapper (*Pagrus auratus*) intake decreased throughout the experiment [21]. However, the main toxin studied then was MTX1, a toxin with different toxicodynamics than CTXs [10]. In experimental models with carnivorous fish such as lionfish, a homogenized food preparation of parrotfish flesh (*Chlorurus microrhinos*) naturally contaminated with CTX was prepared, and a gradual decrease in feeding activity was also observed over the course of the experiment in both CTX-exposed and control fish, although food refusal was greater in the group exposed to CTXs [17]. In fish species naturally exposed to CTX in the wild, constant intake and absence of symptoms have been reported under dietary exposure to ciguatoxin-producing microalgae (*Gambierdiscus polyniensis*) in the species *Naso brevirostris* [25]. This similar behavior in species naturally exposed to CTX in the wild may suggest a physiological adaptation in response to continued ingestion of the toxin. Most of these studies of dietary exposure to ciguatoxins in fish have been performed with juveniles or even larvae, the stage of life in which they have the greatest appetite and in which the energy demand in metabolism increases. In our experiment, all specimens were adults that increased their appetite as they accepted the food provided, showing a food preference factor. The results prompt further investigation into whether individuals adjust their consumption based on the perceived risk associated with ciguatoxin presence.

### 4.2. Biometric Indexes

Biometric indexes in this study provide additional layers to our understanding of the impact of different feeding groups on fish physiology. Despite the absence of significant differences in specific growth rate and condition factor across all feeding groups, group B and the control group stand out with the highest average values in both of these parameters (Table 3). This suggests that, while overall growth and condition factors may not differ significantly among the groups, individual variations within group B are noteworthy. The highest average weight gained in group B, coupled with the highest specific growth rate and condition factor, highlight the potential positive impact of the dietary composition in this group. The observed trends in group B compared with the other groups (group A and control) might indicate a more favorable nutritional environment or that a better utilization of the provided diet may be favored by the palatability of moray flesh. In the study carried out by Benett et al. [19], the growth of *Lagodon romboides* in a ciguatoxic diet compared with a non-toxic one was analyzed; at the end of the experiment, the average weight of the fish fed CTXs was 78.13% and the control group was 65.13%, larger than at the beginning of the study. Low doses of toxin in the tissues after a depuration period was detected, which was related to the growth of the fish in that period; this was a factor in the pseudo-elimination of the toxin. In another study with coral reef fish (*Naso brevirostris*), after 16 weeks of feeding, biomass increased by 400% in both groups (experimental and control), indicating a feeding process without feeding biases [25], as occurred in our study when comparing toxic and non-toxic feeding groups. The control group, characterized by non-toxic feeding, tends to exhibit a lower growth and the lowest condition factor. What we have seen coincides with the response to dietary exposure of ciguatoxin in other experiments mentioned above, where the intake of toxic flesh may not affect the growth of the fish (Table 3). This raises questions about the potential influence of toxic compounds on fish physiology.

The analysis of the hepatosomatic index (HSI) adds another dimension to the discussion. Group B shows the highest average, indicating a potential impact of the dietary composition on liver size, including control and experimental groups from the diet of group B. Moreover, the variations in HSI and GSI between feeding periods suggest temporal dynamics in response to different diets (Table 4). Ramos-Sosa et al. [20] fed 11 goldfish toxic flesh for 43 days and observed a greater HSI in the toxic fish than in the control group without finding significant differences. Featuring another analyzed marine species of the Serranidae family of the grouper, such as *Serranus cabrilla*, Anadon et al. [26] fed seven fish with a concentration of 117.01 mg of *Gambierdiscus* spp. during 10 days, and the study observed how the HSI increased in comparison with the control group; however, in a group of fish fed for 20 days, there was no increase in liver tissue, and tissue even decreased compared with the control group. No significant differences were detected in any cases. However, the lack of significant differences in these comparisons prompts further exploration into the factors influencing hepatic and gonadal conditions.

In the groupers of the present study, all females in the first stage of reproductive development had a total length of 42.06 ± 5.27 cm; it is known that the reproductive biology of the grouper is protogynous hermaphrodite, and they reach first sexual maturity as females at 5 years of age [45,46]. The results of the experiment showed the lowest GSI in dietary group B, both control and experimental, and greater liver development in this group, which could suggest a different response to the diet in group A, which presented a higher GSI. Therefore, in group A of dietary exposure, prolonged exposure to the ingestion of flesh contaminated with ciguatoxin, it was not observed that the stage of reproductive development affected gonadal development. However, group B, despite showing higher SGR, CF, and HSI values in a shorter study time than group A (6 and 12 weeks), had a GSI that was lower during this period compared with group A, which showed a higher GSI index (0.60 and 0.67). These variations can be explained considering the growth of the specimens and the use of energy consumed. In other studies [27,28], it has been observed how prolonged ingestion of CTX1B can cause reproductive problems related to gonadal development, egg production, and hatching in *Oryzias melastigma*. Additionally, there are several experimental models regarding this aspect, focused on larvae of this same fish species, and anomalies have been observed in the column, impeding the ability to orient and increasing mortality [27,47,48]. Also, in recent studies with *O. melastigma*, embryos that have been injected with purified Pacific ciguatoxin-1 (CTX1B) induced detrimental effects during embryonic development [23]. And, working with the same species, a greater accumulation of CTX1B could be quantified in females (24.1 pg ± 1.4%) than in males (9.9 pg ± 0.4%), proving that dietary exposure to ciguatoxin affects the survival of offspring and causes maternal transfer of the toxin [23]. In the dietary exposure experiment in goldfish (*Carassius auratus*) fed C-CTX1-contaminated flesh by Sanchez-Henao et al. [18], fish in the control group showed gonadal development and courtship behaviors, while fish with toxic food did not show reproductive behaviors. It should be noted that these are experimental models in which anomalies in gonadal, embryonic, and reproductive development were detected due to toxic feeding that are not usually present in the marine environment exposed to ciguatoxins. Possibly, these fish do not show the same physiological adaptations as other species exposed to marine biotoxins in the environment, such as the groupers.

### 4.3. Fish Behavior

The meticulous observation of abnormal behavioral signs, including swimming patterns, food-related responses, interaction with caregivers, and respiratory patterns, on a daily basis provides valuable insights into the potential impacts of the experimental diets. The absence of abnormal signs in any of the recorded fish suggests that, at a visible and behavioral level, the experimental groups did not exhibit overt signs of distress or discomfort. The lack of food refusal or attraction, abnormal interactions, and unusual respiratory patterns indicate a level of adaptability or tolerance to the dietary conditions imposed during the study. Observation of swimming with video cameras is a good tool for determining changes that can be seen visually, but it is rarely used in feeding experiments with CTXs. Li et al. [28] performed recordings of fish (*Oryzias melastigma*) fed *Artemia metanauplii* previously exposed to *G. caribaeus*. Experimental fish reduced swimming speed and acceleration and encountered loss of balance and transient vertical swimming compared with the control group. To the best of our knowledge, our observation criteria were different in this case; as the adult grouper used is a short-swimming species, it does not swim with rapid or accelerated movements, which is why the movement of the fins, opening of the operculum, changes in coloration, positioning in the tank, and arrangement of the dorsal spines may be more appropriate criteria for our study species. However, there are other studies that have detected abnormal signs in the swimming and behavior of fish without video recordings. Sanchez-Henao et al. [18], when working with goldfish, found behavioral disturbances and signs of intoxication after 15 days of toxin feeding. Fish showed lethargy and color brightness alteration in previous studies by Ledreux et al. [24], where symptoms were also detected in *Mugil cephalus*. These two species studied (*M. cephallus* and *C. auratus*) are herbivorous and omnivorous fish, respectively, and are not exposed to ciguatoxins in the wild.

Moreover, in two different early dietary exposure studies by Davin et al. [49] featuring carnivorous fish, secondary consumers of the food chain such as *Thalassoma bifasciatum* were fed freeze-dried *Gambierdiscus* spp. cells and showed symptoms such as loss of balance and orientation, erratic swimming, changes in skin color, and sudden movements. In the other study by Davin et al. [50], also with piscivorous fish, three marine fish (*Epinephelus fulvus*, *Lutjanus apodus*, and *Lutjanus mahogoni*) and a freshwater fish (*Micropterus salmoides*) were fed homogenized barracuda flesh naturally contaminated with CTX and, in a separate experiment, *M. salmoides* with lyophilized *Gambierdiscus* spp. cells. All experimental fish species showed symptoms, while the control group remained asymptomatic. The first symptom reported was loss of appetite after ingesting 0.6 mg of the extract; at a lower concentration of toxin, the fish took longer to lose appetite. Other symptoms observed after several exposures were disorientation and positioning themselves upside down, although they regained their position by swimming. In addition, the fish lost their normal coloring, turning completely brown. As in our experiment, there was also a decrease in intake related to the toxic feeding of group A; therefore, loss of appetite was also reported. However, we did not detect sudden movements, and these fish were quite sedentary and presented rapid changes in coloring patterns.

According to the observations of our experiment, we could suggest that under these feeding and experimental conditions of the fish subjected to CTX treatment, we have not found evidence of changes in behavior or symptoms that affect the health and wellbeing of the dusky grouper. Both experimental groups (A and B) and the control group had the same response to dietary exposure in terms of behavioral signs. The results of other studies that contrast with ours could respond to differences in the experimental diet, either in the matrix of the flesh used for feeding or in the content of the toxin used; fish could perhaps develop symptoms in higher doses than those used in our study. Furthermore, the characteristics of the species used can also suggest differences in the behavior of the specimens.

On the other hand, the inclusion of quantitative measures while watching fish, such as the counting of pectoral and dorsal fin movements, adds an additional layer of objectivity to the analysis. The lack of significant differences in fin movement counts between fish in different dietary groups suggests that, at least at a visible and quantifiable level, there were no pronounced alterations in the locomotor or exploratory behaviors of the fish. However, it is important to note that while visible signs may not be apparent, there could still be subtle physiological or internal changes that are not immediately evident through visual observation.

### 4.4. Hematocrit and Blood Biochemistry

The assessment of hematocrit and blood biochemistry in this study provides insights into the physiological responses of the experimental groups to different dietary regimes.

Starting with hematocrit values, an interesting observation is the higher values in the last sampling of experimental group A compared with other dietary groups. While non-statistically significant differences were detected, this trend prompts further investigation into the potential factors influencing hematocrit levels, such as dietary components or exposure duration. The decrease in the average of red blood cells and white blood cells in the last sampling period (18 weeks) compared with the previous ones (4 weeks and 10 weeks) adds a temporal dimension to the analysis, suggesting potential dynamics in blood cell composition that merit further exploration. This observation may suggest changes in physiology and a negative impact on the immune system with respect to the time of exposure of the toxin. There are few studies related to the study of hematocrit and blood biochemistry values in fish and their exposure to ciguatoxins. This decrease in white blood cells may indicate a negative effect on the immune system due to the diet. In addition, parasitological studies have been carried out in groupers subjected to health control by the Ciguatera Control Program in the Canary Islands [51]; in that study, the prevalence of parasite larvae *Pintneriella* sp. of was 96.4%, and the high percentage of parasitosis in these fish with CTXs may suggest that the weakness of the immune system in these fish favors parasitic infestations.

Intrinsic and extrinsic factors can influence blood parameters in fish, such as fish species, stress, environmental factors, and nutritional status. Fluctuations in hematological biochemical parameters in fish can be used as important tools for determining alterations in fish metabolism [31]. Thus, although the absence of statistical differences between dietary regimes for GLU, PRO, LACT (Table 5), TRIG, and CHOL (Figure 3 and Figure 4) is notable, the higher levels of TRIG and total CHOL in fish from control group compared with those fed toxic flesh raises questions about the impact of CTXs on lipid metabolism; moreover, the low dispersion of the data suggest the interconnectedness of lipid profiles in response to different diets. In the study by Gonzalez et al. [29], the morphological alterations in the liver due to the ingestion of *Gambierdiscus* spp. in the *S. cabrilla* were particularly studied, and they observed an accumulation of lipid droplets in the liver cells caused by the dose of *Gambierdiscus* cells and its prolonged ingestion. Ciguatoxins are fat-soluble compounds, and they can cause alterations in fatty acids; lipids can accumulate in hepatocytes, and these marine biotoxins can modify lipid metabolism [26].

Lactate levels, while showing the highest measurements in experimental group A at the end of the exposition period, did not exhibit significant differences compared with the other groups. This suggests that lactate levels may be influenced by factors beyond dietary composition, requiring a more comprehensive exploration of contributing variables. Lactate is a metabolic index of stress as a secondary stress response. In situations of acute stress, lactate in the blood increases due to an increase in energy demand via anaerobic means [30]. This result may suggest a stress factor due to the time of exposure to the toxic diet. In the study performed by Ramos-Sosa et al. [20], in which the authors analyzed the hepatic metabolism of glucose, lactate, and other metabolites such as alanine and taurine after feeding flesh with CTX to goldfish, they observed that the glucose–alanine cycle was altered; they also found an increase in hepatic glycogen in fish with toxic feeding, which suggested a greater HSI and glycogen reserves in the liver, which supports our results, where we found a greater HSI in the last weeks of feeding and an increase in lactate levels.

The analysis of liver enzymes also provides valuable information on hepatic function. While significant differences were found only in AST/GOT (Figure 5), the progressively increasing values of both AST/GOT and ALP (Figure 6) in fish from experimental group A warrant attention. In contrast, group B did not exhibit statistically significant differences in liver enzyme levels when comparing the experimental and control groups. This divergence in hepatic responses between groups A and B and the control group raises questions about the differential effects of toxic load and non-toxic feeding on liver function, underscoring the need for further exploration. In this section, we can contrast the results with the experiment by Anadon et al. [26], who studied alterations in the enzymology of the liver of juveniles *S. cabrilla* under the conditions of feeding with *Gambierdiscus* spp. with a concentration of 2800 cells/mg of extract; the authors found that phosphatase enzymes showed significant differences in their concentration, and specifically ALP was higher in intoxicated fish after dietary exposure for 20 days. They suggest an increase in this enzyme due to liver damage as a result of hepatocellular lesions that they observed at the histological level. In the article by Li et al. [28], the authors sequenced genes involved in the synthesis of fatty acids in the liver and observed that in fish exposed to higher doses of toxin, these genes were inhibited; at low levels of toxin, these genes were activated. High amounts of toxin generate a risk of apoptosis due to liver damage, which may explain the alteration in AST and ALP levels. As observed in the current study, in dusky groupers exposed to a greater amount of CTX over time, lipid metabolism has been altered (low levels of TRIG and CHOL); this may be related to hepatic metabolism, where an increase in the HSI was determined, as well LACT and GLU levels, which coincides with the other results of the authors presented above.

The size population used in this study could preclude strong conclusions; however, the tendencies observed and discussed suggest that the ingestion of ciguatoxins in dusky groupers under these laboratory conditions can favor weakening of the immune system, an increase in secondary parameters of stress, and also alteration in hepatocytes and fatty tissues and changes in the transformation of fatty acid levels in the systemic circulation, which can result in enzymatic alterations, particularly in AST/GOT and ALP. These metabolic alterations could involve possible liver damage in dusky grouper, which is a species naturally exposed to CTX in the trophic web. Contrarily, the functional changes described in the current study have not produced any visible symptoms in the exposed fish.

## 5. Conclusions

In conclusion, the observed trends in food intake hematocrit, blood cell counts, plasma biochemical parameters, and liver enzymes highlight the complexity of the interactions between CTXs in diet and physiological markers. The nuanced variations and temporal dynamics suggest a need for comprehensive investigations to unravel the underlying mechanisms driving these responses.

Prolonged ingestion of toxins over time may reduce the appetite of the specimens studied, and their tolerance to toxic feeding have not given rise to symptoms in their behavior or swimming. It is possible that at a higher dose of toxic exposure they may present symptoms, but further hepatotoxic and histological studies are necessary to know the damage to the liver, as we have observed changes in the physiology of fish exposed to CTX.

## Figures and Tables

**Table 1:** Fish sampling carried out in the experimental and control groups during this trial.

Groups	Sampling Week	Fish per Sampling (n)
A ^1^	4, 10, 18	2
B ^2^	6, 12	2
Control group ^3^	0 *, 12, 18	1

^1^ Group fed ciguatoxic amberjack flesh. ^2^ Group fed ciguatoxic moray eel flesh. ^3^ Group fed negative to CTX-like toxicity flesh. * At this point, two fish were sampled.

**Table 2:** Average intake of each experimental group divided between the beginning of the experiment and the end, with each period represented by sampling.

Feeding Periods ^1^	Feeding Days per Sampling ^2^	Intake (%) ^3^
Group A		
0–4 (*n* = 6)	174	1.06 ± 0.14
4–10 (*n* = 4)	168	0.87 ± 0.18
10–18 (*n* = 2)	108	0.74 ± 0.03
Group B		
0–6 (*n* = 4)	168	1.18 ± 0.08
6–12 (*n* = 2)	72	1.00 ± 0.23

^1^ Feeding periods represented in the sampling weeks. ^2^ Total days of feeding in each period. ^3^ Average intake.

**Table 3:** Average biometric indexes by dietary group.

Groups	SGR (% day^−1^) ^1^	CF (%) ^2^	Weight Gained (g) ^3^
A (*n* = 6)	0.18 ± 0.10	2.28 ± 0.33	157.8 ± 85.8
B (*n* = 4)	0.21 ± 0.18	2.33 ± 0.18	218.5 ± 263.6
Control, A and B (*n* = 4)	0.14 ± 0.16	2.26 ± 0.31	241.0 ± 272.9

^1^ Specific growth rates by dietary group. ^2^ Condition factor by dietary group. ^3^ Weight gained throughout the experiment per dietary group.

**Table 4:** Average morphometric indexes by sampling week.

Sampling Week	Group ^1^	HSI ^2^	GSI ^3^
4	A	0.94 ± 0.51	0.39 ± 0.13
10		0.96 ± 0.17	0.60 ± 0.22
18		1.19 ± 0.06	0.67 ± 0.06
6	B	1.17 ± 0.2	0.39 ± 0.1
12		0.99 ± 0.07	0.35 ± 0.16
12, 18	Control (A and B)	0.99 ± 0.12	0.46 ± 0.16

^1^ Group of dietary exposure. ^2^ Hepatosomatic index. ^3^ Gonadosomatic index. *n* = 2 fish per sampling.

**Table 5:** Average biochemical parameters in plasma by dietary group.

Groups	Glucose (mg/dL)	Lactate (mg/dL)	Protein (mg/dL)
A (*n* = 6)	321.02 ± 424.1	22.4 ± 28.1	201.8 ± 114.1
B (*n* = 4)	58.59 ± 27.6	6.9 ± 1.4	127.3 ± 111.8
Control, A and B (*n* = 3 *)	166.1 ± 155.3	15.8 ± 11.9	317.7 ± 128.9

* One of the specimens could not be analyzed.

## Data Availability

The data presented in this study are available on request from the corresponding author.
